# An *Ustilago maydis* chassis for itaconic acid production without by‐products

**DOI:** 10.1111/1751-7915.13525

**Published:** 2019-12-27

**Authors:** Johanna Becker, Hamed Hosseinpour Tehrani, Marc Gauert, Jörg Mampel, Lars M. Blank, Nick Wierckx

**Affiliations:** ^1^ Institute of Applied Microbiology (iAMB) Aachen Biology and Biotechnology (ABBt) RWTH Aachen University Worringerweg 1 Aachen 52074 Germany; ^2^ BRAIN AG (Biotechnology, Research and Information Network) Darmstädter Str. 34‐36 Zwingenberg 64673 Germany; ^3^ Institute of Bio‐ and Geosciences IBG‐1: Biotechnology Forschungszentrum Jülich Jülich 52425 Germany

## Abstract

*Ustilago maydis* is a promising yeast for the production of a range of valuable metabolites, including itaconate, malate, glycolipids and triacylglycerols. However, wild‐type strains generally produce a potpourri of all of these metabolites, which hinders efficient production of single target chemicals. In this study, the diverse by‐product spectrum of *U. maydis* was reduced through strain engineering using CRISPR/Cas9 and FLP/FRT, greatly increasing the metabolic flux into the targeted itaconate biosynthesis pathway. With this strategy, a marker‐free chassis strain could be engineered, which produces itaconate from glucose with significantly enhanced titre, rate and yield. The lack of by‐product formation not only benefited itaconate production, it also increases the efficiency of downstream processing improving cell handling and product purity.

## Graphical abstract

In this study, the diverse by‐product spectrum of *U. maydis* – including malate, glycolipids, and triacylglycerols – was reduced through strain engineering using CRISPR/Cas9 and FLP/FRT, greatly increasing the metabolic flux into the targeted itaconate biosynthesis pathway. With this strategy, a marker‐free chassis strain could be engineered which produces itaconate from glucose with significantly enhanced titre, rate, and yield. The lack of by‐product formation not only benefited itaconate production, it also increases the efficiency of downstream processing improving cell handling and product purity.

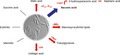

## Introduction

*Ustilago maydis*, a member of the Ustilaginaceae family, is a well‐established model organism for the investigation of fungal mating, DNA recombination, RNA biology, cell signalling and plant‐pathogen interactions (León‐Ramírez *et al.*, 2014). It also has large biotechnological potential due to features such as natural production of a wide range of value‐added molecules, insensitivity to medium impurities and hydromechanic stress, and unicellular growth (Klement *et al.*, 2012; Klement and Büchs, 2013; Maassen *et al.*, 2014). Main products include organic acids (malate, succinate, itaconate, itatartarate and (S)‐2‐hydroxyparaconate), polyols (erythritol and mannitol), glycolipids (mannosylerythritol lipids and ustilagic acid), intracellular triacylglycerols (Guevarra and Tabuchi, 1990; Bölker *et al.*, 2008; Moon *et al.*, 2010; Feldbrügge *et al.*, 2013; Aguilar *et al.*, 2017) and proteins (Kämper *et al.*, 2006; Mueller *et al.*, 2008; Geiser *et al.*, 2013). Culture conditions influence the composition of the product spectrum. For instance, nitrogen starvation is an important activator for the synthesis of organic acids and glycolipids (Hewald *et al.*, 2005, 2006; Teichmann *et al.*, 2007). A decrease in pH however leads to a switch from the production of organic acids to polyols and glycolipids as preferred metabolite classes (Geiser *et al.*, 2014).

Itaconate is an established bio‐based product with high potential as platform chemical. This chemical and its derivatives already have a widespread application spectrum, for example in the industrial production of fibres, plastics, rubbers and surfactants, but also as bioactive compound in the agriculture, pharmacy or medical sectors (Willke and Vorlop, 2001; Okabe *et al.*, 2009; Betancourt *et al.*, 2010; Bera *et al.*, 2015; De Carvalho *et al.*, 2018; Kuenz and Krull, 2018). The market for itaconic acid is expected to expand in the next years (Weastra, 2012). So far, this chemical is commercially produced in fermentation processes using the filamentous fungus *Aspergillus terreus*. Other natural producers like *U. maydis*, *Candida* sp. or *Rhodotorula* sp. are to date not competitive with the production parameters of *A. terreus* that is able to reach itaconate titres of up to 160 g l^−1^, yields of 0.63 g_ITA_ g_Glu_^‐1^ and rates of 1.53 g l^−1^ h^−1^ (Steiger *et al.*, 2013; Krull *et al.*, 2017). Nevertheless, *A. terreus* has a high sensitivity to fermentation conditions such as medium impurities, hydro‐mechanical stress, viscosity of fermentation broth or oxygen supply (Klement *et al.*, 2012; Kuenz *et al.*, 2012). Morphology control is a major challenge which hinders reproducibility of cultivations, even when they are performed with the same strains (Willke and Vorlop, 2001; Kuenz *et al.*, 2012).

Therefore, research increasingly focuses on Ustilaginaceae as alternative unicellular biocatalysts. *U. maydis* represents a promising organism for application in industrial itaconate production. Recent efforts have achieved considerable improvements in itaconate production with *Ustilago*, including characterization and upregulation of the itaconate gene cluster and associated pathway (Geiser *et al.*, 2016b,c, 2018), engineering of transporters involved in itaconate production (Hosseinpour Tehrani *et al.*, 2019b), and morphological and metabolic engineering of the acid‐tolerant *U. cynodontis* (Hosseinpour Tehrani *et al.*, 2019c). However, the potpourri of metabolites naturally synthesized by *U. maydis* results in suboptimal specificity, productivity and yield of itaconate as a product. These impurities hinder downstream purification of itaconate as the main product by for example co‐precipitation and co‐crystallization and can also impede prior biomass separation unit operations such as settling or centrifugation (Regestein *et al.*, 2018). This issue can be addressed by metabolic engineering, which is enabled by a strong basis of genomic tools and data, including a well‐annotated genome sequence (Kämper *et al.*, 2006) as well as shotgun sequences of related itaconate producing Ustilaginaceae (Geiser *et al.*, 2016a, 2018). Engineered promoters enable strong heterologous gene expression under a range of conditions (Bottin *et al.*, 1996; Brachmann *et al.*, 2001; Flor‐Parra *et al.*, 2006; Zarnack *et al.*, 2006; Sarkari *et al.*, 2014; Zambanini *et al.*, 2017), and *in vivo* and *ex vivo* sensors enable high‐resolution monitoring of a range of cellular factors (Büchs, 2001; John *et al.*, 2003; Anderlei *et al.*, 2004; Hartmann *et al.*, 2018). For efficient gene deletion, gene insertion, and promoter exchange, different genetic tools are available, including CRISPR/Cas9 genome editing (Schuster *et al.*, 2016), FLP/FRT system with marker recycling (Khrunyk *et al.*, 2010) or Golden Gate Cloning (Terfrüchte *et al.*, 2014).

Previous studies already achieved promising results in characterizing and engineering product formation pathways. Regarding itaconate production, a combination of deleting *cyp3*, which encodes an itaconate oxidase that converts itaconate into 2‐hydroxyparaconate, and overexpressing *ria1*, which encodes the itaconate gene cluster regulator, increased itaconate titres by nearly 4‐fold (Geiser *et al.*, 2016b). This also led to a strong decrease in malate production due to upregulation of *mtt1* encoding a *cis*‐aconitate/malate antiporter (Geiser *et al.*, 2016b; Scarcia *et al.*, 2019). MEL biosynthesis is mediated by a gene cluster comprising five genes, and single mutant strains *U. maydis* MB215 ∆*emt1*, ∆*mac1* and ∆*mac2* completely lost their ability to produce MELs (Hewald *et al.*, 2005, 2006). The gene cluster enabling UA biosynthesis is formed by 12 open reading frames including the cytochrome P450 monooxygenase encoded by *cyp1* (Hewald *et al.*, 2005; Teichmann *et al.*, 2007, 2010). Intracellular triacylglycerol (TAG) formation has not been studied in detail in *Ustilago*, but in other oleaginous species, diacylglycerol acyltransferases (DGAT) represent main contributors by mediating the final acylation of diacylglycerols to TAGs (Guo *et al.*, 2009; Aguilar *et al.*, 2017).

**Figure 1:**
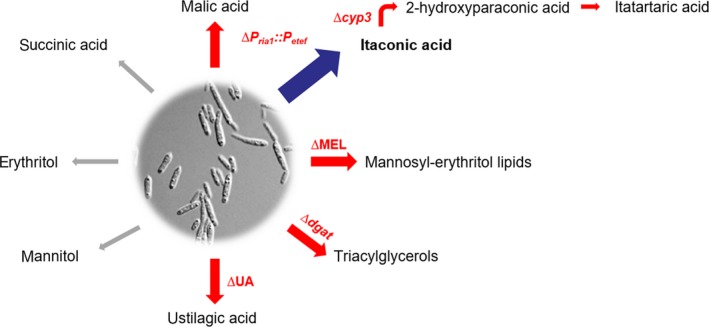
Overview of known *U. maydis* MB215 metabolites. The targets addressed by metabolic engineering are labelled in red, the main product itaconic acid in blue. Grey arrows indicate metabolites that were not detected under the conditions tested in this study.

Based on this detailed methodical and biochemical knowledge, this study reduces the product spectrum of *U. maydis* through marker‐free gene cluster deletion and promoter replacement (Fig. 1). This successfully focused most of the carbon flux into itaconate as main product, while simultaneously generating a chassis that is more manageable in a process context.

## Results and discussion

### Insights into the genome sequence of an *U. maydis* deletion strain engineered for an improved itaconate production

To generate an itaconate producing *U. maydis* chassis with reduced by‐product formation, multiple knockouts were successively performed in one strain. Due to a limited number of selection markers suitable for *U. maydis*, and considering the benefit of marker‐free strains in an industrial biotechnology setting, two cloning techniques were used to generate a marker‐free strain: CRISPR/Cas9 (Schuster *et al.*, 2016) and marker insertion by homologous recombination with subsequent FRT‐FLP‐mediated marker removal (Khrunyk *et al.*, 2010). When using the Cas9‐based system, repair templates were used for all knockouts in order to achieve full gene deletions through homologous recombination. These templates were transformed along with the Cas9 plasmid which included the guide RNA of the target gene. Primers for construction of all associated sequences and the corresponding plasmids are listed in Tables S2 and S3.

**Figure 2:**
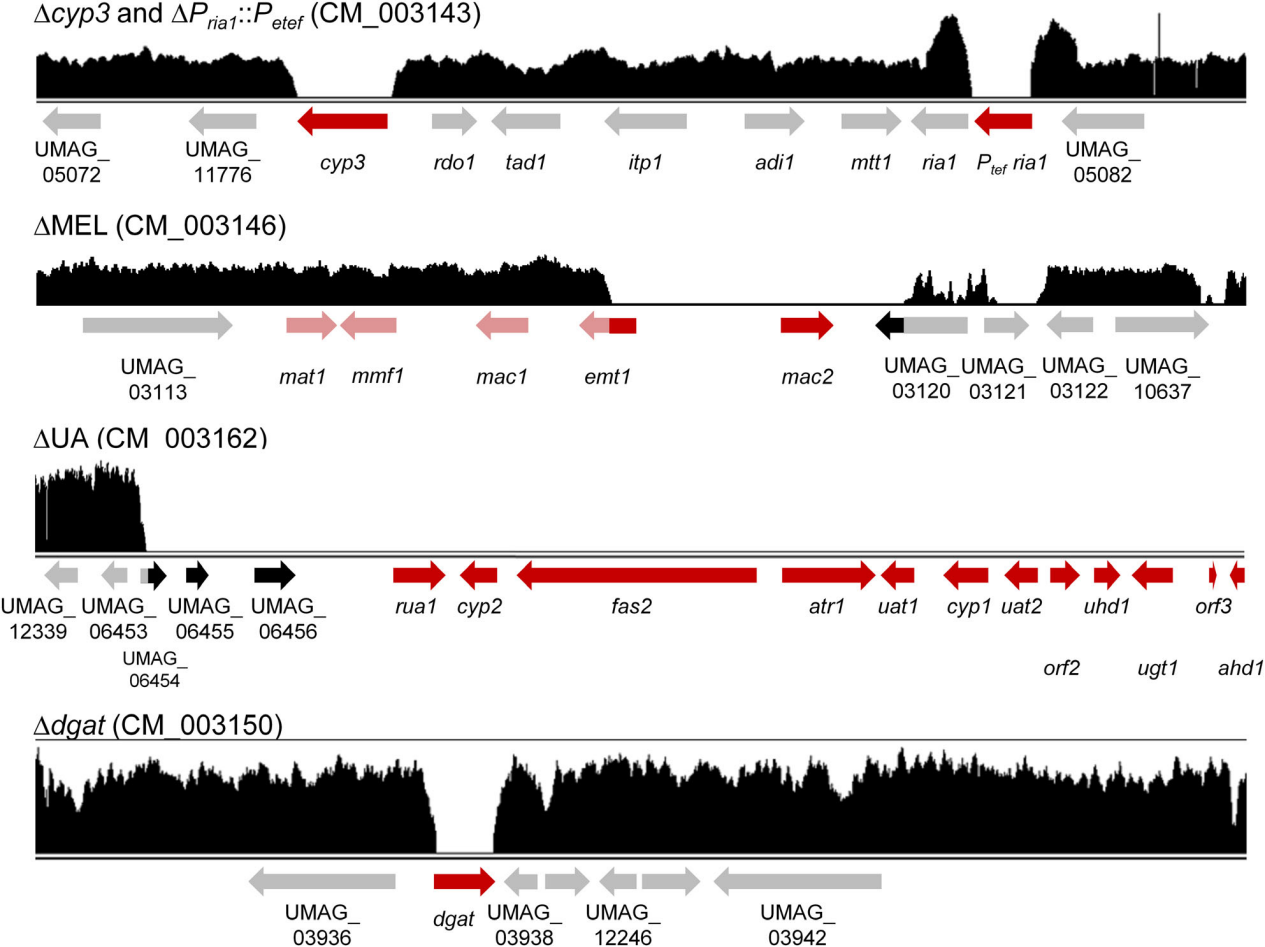
Genome analysis of the ITA chassis strain *U. maydis* MB215 ∆*cyp3* ∆MEL ∆UA ∆*dgat* ∆*P_ria1_*::*P_etef_* by whole‐genome sequencing. The coverage of Illumina sequencing reads mapped against the *U. maydis *521 wild‐type sequence visualizes the different deletion loci. Targeted genes are marked in red, verified deletions in dark red and unexpectedly non‐performed deletions in light red. Further unexpected gene deletions are marked in black.

The conversion of itaconate into (S)‐2‐hydroxyparaconate was abolished by the deletion of *cyp3* (Geiser *et al.*, 2016b). To stop glycolipid production, repair templates were designed to remove the whole gene clusters for MEL (Hewald *et al.*, 2006) and UA (Teichmann *et al.*, 2007) biosynthesis. The production of TAGs, forming intracellular lipid droplets as main energy storage in *U. maydis* (Aguilar *et al.*, 2017), was reduced by single gene deletion of UMAG_03937. This gene, subsequently named *dgat*, encodes a putative diacylglycerol acyltransferase based on sequence alignment of DGAT2 from *Saccharomyces cerevisiae* (Liu *et al.*, 2011). As last modification, *ria1*, encoding the itaconate cluster regulator, was overexpressed by a promoter exchange to improve itaconate and decrease malate production (Geiser *et al.*, 2016b). This was achieved by the inclusion of the *P_etef_* promoter into the repair template yielding a marker‐free insertion (Hosseinpour Tehrani *et al*., 2019a). For the generation of ∆*cyp3*, ∆MEL, ∆UA and ∆*P_ria1_*::*P_etef_*, the CRISPR/Cas9 system was used, while ∆*dgat* was achieved using the FRT/FLP‐mediated approach. All modifications were performed consecutively in the order ∆*cyp3*, ∆MEL, ∆UA, ∆*dgat* and ∆*P_ria1_*::*P_etef_*, ultimately yielding the final chassis strain *U. maydis* MB215 ∆*cyp3* ∆MEL ∆UA ∆*dgat* ∆*P_ria1_*::*P_etef_* (hence called *U. maydis* MB215 ITA chassis). While ∆*cyp3*, ∆*dgat*, and ∆*P_ria1_*::*P_etef_* could successfully be verified by PCR analysis, the confirmation of the deletion of larger genomic fragments, such as the 18.5 kb MEL cluster (Hewald *et al.*, 2006) and the 58 kb UA cluster (Teichmann *et al.*, 2007), could not be conclusively confirmed this way. Resequencing of the chassis strain genome and comparison to the reference genome of *U. maydis *521 wild type (Kämper *et al.*, 2006) confirmed the successful deletion of *cyp3* and *dgat*, but also exposed several unexpected phenomena with the other modifications (Fig. 2). As expected, no reads mapped against the native *ria1* promoter region, indicating successful deletion of the 1334 bp fragment. The coverage of reads mapped against the native *P_tef_* promoter was almost twice as high as the baseline (not shown), indicating that the stronger *P_etef_* promoter was successfully inserted. However, the coverage of the two 1000 bp regions flanking the promoter, precisely matching the homology arms of the repair template, is approximately twice the mean value. Further single read analysis and PCR verification indicated that the entire pJET1.2‐vector carrying the repair template had been integrated into the genome (Fig. S1). Thereby, both flanks exist as duplicates, in the native and in the synthetically generated form. This led to a duplication of *P_etef_ ria1*, with one truncated 985 bp *ria1* and one full 1231 bp gene. Although it is unclear if this truncated version is active, it is unlikely that further overexpression of Ria1 increases itaconate production, given that separate experiments aimed at increasing Ria1 expression through multicopy insertion of a *P_etef_ria1* construct did not increase production compared to the single copy insertion strain reported by Geiser *et al.* (2016c) (Bator, 2017, unpublished). The duplication of the *ria1* flanks comes with a slight risk of genetic instability, although this would equally be the case for the abovementioned insertion of *P_etef_ria1* into the CBX locus. The chance of duplication can be avoided in the future through the use of a linear PCR‐generated repair template.

The aspired deletion of the 18.5 kb MEL gene cluster identified a disadvantage of the Cas9 system. The system is based on a Cas9 nuclease cleaving within a specific target gene, in this case *emt1*. The formed double strand break was supposed to be repaired via homologous recombination using the repair template specifically designed for the deletion of the MEL cluster. However, the sequencing shows only the successful deletion of the complete *mac2* gene and of the first 43% of the *emt1* sequence, while the other cluster genes are still present. The adjacent UMAG_03120 gene might also be partially deleted, although coverage was generally poor in this region. Even though the full cluster was not deleted as intended, the deletion of *mac2* and the disruption of *emt1* are sufficient to abolish MEL synthesis in *U. maydis* (Hewald *et al.*, 2005, 2006). A similar effect occurred with the deletion of the 58 kb UA biosynthesis gene cluster. Besides the targeted elimination of all UA cluster genes, additional genes upstream of the cluster were also deleted. UMAG_06454, UMAG _06455 and UMAG _06456 encode hypothetical proteins with unknown function. This event of co‐elimination of adjacent sequences also occurred for a part of gene UMAG_03120 flanking the MEL cluster and encoding an uncharacterized protein. No significant sequence similarities between the repair template and sequences surrounding the affected regions were found. A lack of reads in the end of chromosome 23 where the UA cluster is encoded was also noticeable. However, this is likely a mapping artefact, as the coverage of the telomeric regions of other chromosomes exhibited an overall downgraded coverage quality in these highly repetitive sequences.

Summarized, all intended genomic modifications were achieved but in three out of four cases where the Cas9 system was used unintended side‐effects occurred. These unintentional effects indicate that homologous recombination often fails to resolve along the designed repair templates, even with long 1 kb flanks. Although it is currently unclear what causes this, it could possibly be avoided in the future by using higher concentrations of linear repair template with longer flanks for recombination. In the case of deletion of longer genomic stretches, the unpredictability of the chosen method is exacerbated by the fact that correct deletions are harder to detect by PCR methods due to the lack of a suitable positive control. This highlights the usefulness of genome resequencing, especially in this case of multiple iterative deletions. Although unexpected deletions did occur, these had no observable negative effect on the fitness of the strain, as described below.

### Detailed analysis of lipid production in *U. maydis* ITA chassis and its progenitors

**Figure 3:**
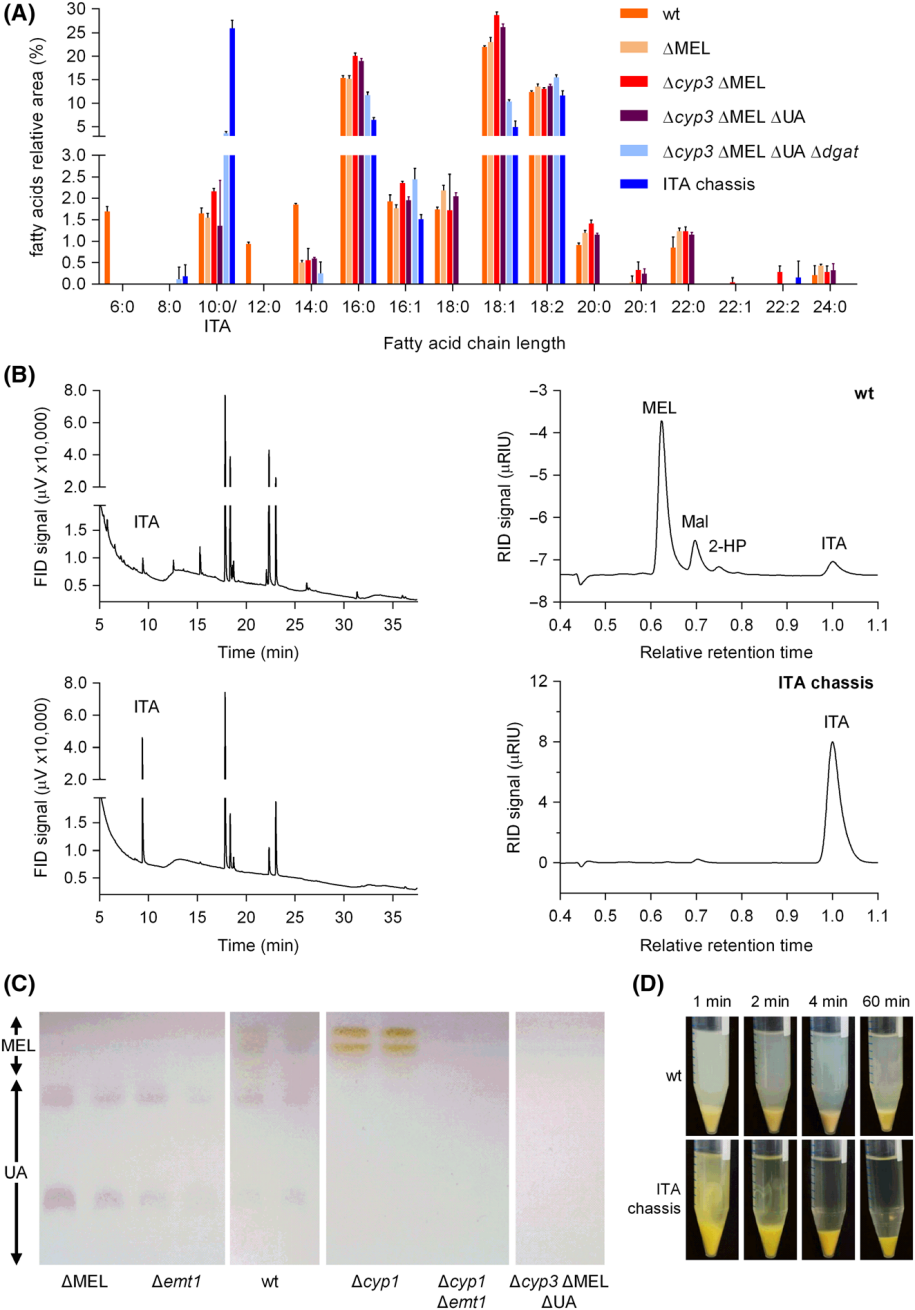
Lipid analysis of *U. maydis* MB215 wild type and various deletion strains analysed by fatty acid methyl esters (FAMES) detection. For lipid formation, all strains were incubated for 72 h in screening medium with 50 g l^−1^ glucose and a pH of 6.5 buffered with 100 mM MES using 24‐well System Duetz^®^ plates. Error bars indicate the standard error of the mean (n = 6). A. Relative abundance of fatty acids with different chain length. The peak of dimethyl itaconate overlaps that of the C_10:0_ FAMES. B. Representative FAMES and HPLC diagrams of the wild type and ITA chassis strain. In the FAMES spectrum, retention time correlates with chain length. For HPLC analysis, supernatants were obtained from culture broths after 96 h (*U. maydis* MB215 wild type) and 124.5 h (ITA chassis) of cultivation and retention times are shown relative to itaconic acid. MEL: mannosylerythritol lipids, Mal: malate, 2‐HP: (S)‐2‐hydroxyparaconate, ITA: itaconate. C. Thin‐layer chromatography of ethyl acetate extracts of duplicate cultures of *U. maydis* MB215 wild type, ∆MEL, ∆*cyp3* ∆MEL ∆UA and ∆*emt1* and/or ∆*cyp1* controls (Hewald *et al*., 2006). The brightness of photos was adjusted to enhance comparability between samples. D. Impact of lipidic by‐product deletion on the centrifugation efficiency (5000 *g*, 5 ml culture broth) of the wild type and ITA chassis.

The lipid spectra of the deletion strains were quantitatively characterized by GC‐FID analysis of fatty acid methyl esters (FAMES). Lipidic extracts were obtained from the whole culture broths after 72 h of cultivation (screening medium, 50 g l^−1^ glucose, pH 6.5 buffered with 100 mM MES, System Duetz^®^) in order to quantify both intra‐ and extracellular lipids. The ITA chassis and all engineered precursor strains were analysed along with the wild type in order to identify the impact of each single mutation (Fig. 3A).

The molecular structures of the three investigated lipid classes (MELs, UA and TAGs) feature different fatty acid chain length profiles: two short‐ to medium‐chain (C_2_ to C_14_) fatty acids for MELs (Hewald *et al.*, 2006; Huang *et al.*, 2011), one short‐chain (C_6_ or C_8_) and a C_16_ fatty acid for UA (Teichmann *et al.*, 2007, 2011), and three long‐chain fatty acids, predominantly C_16_ and C_18_, for TAGs (Aguilar *et al.*, 2017). As expected from the molecular structures, the deletion of glycolipid production, especially ∆MEL, led to a drastic reduction or even complete absence of C_6_, C_12_ and C_14_ fatty acids (Fig. 3A). Consequently, a general shift in the fatty acid production spectrum was observed, represented by an increased production of long‐chain fatty acids, indicating a fostered TAG synthesis by glycolipid elimination. The subsequent deletion of *dgat* resulted in a significant reduction or complete loss of these long‐chain fatty acids. The production of C_18:0_ as well as the whole spectrum of C_20_ to C_24_ fatty acids was completely eliminated, while C_16_ and C_18:1_ fatty acids were reduced by 38, respectively, 60% in comparison to the values of the precursor triple mutant. This confirms that the putative *dgat* (UMAG_03937) plays a major role in TAG production. With the final overexpression of *ria1* included, the resulting *U. maydis* MB215 ITA chassis produced 15.7‐fold more itaconate than the wild type. Simultaneously, the synthesis of C_16_ and C_18:1_ fatty acids was reduced by further 38–52% in this strain compared to its predecessor. These reduction effects in lipid production are likely due to an increased flux towards itaconate, which may impose a ‘pull’ effect on the competing lipid synthesis pathways.

The elimination of lipidic by‐product formation is also clearly apparent from the corresponding GC‐chromatograms (Fig. 3B; Fig. S3). Whereas the wild‐type samples have a multitude of small peaks, the chromatogram of the ITA chassis is completely smooth aside from the large dimethyl itaconate peak and the C_16_/C_18_ peaks associated with the membrane lipids from the biomass. The absence of glycolipids was also confirmed by thin‐layer chromatography (Fig. 3C; Teichmann, 2009; Geiser *et al.*, 2014).

In all, the modifications engineered into the ITA chassis greatly reduced the production of lipidic products in this strain. The elimination of these by‐products itself had a significant positive effect on itaconate production, which was amplified by the overexpression of the itaconate gene cluster regulator encoded by *ria1*.

Since the GC‐FID FAMES analysis is limited to the detection of compounds with a carboxylic acid group able to be methylated, supernatants of the strains mentioned above were additionally analysed by HPLC‐RI (Fig. 3B). This method allows the detection of sugars and polyols and enables the analysis of organic acids in a more quantitative manner. In contrast to the wild type and ITA chassis strains, all knockout strains were unable to consume the initial glucose concentration of 50 g l^−1^ within 96–124 h, resulting in a glucose peak at a relative retention time (rRT) of 0.64 corresponding to glucose concentrations between 0.90 ± 0.2 and 7.0 ± 0.4 g l^−1^. This supports the hypothesis of a metabolic ‘pull’ effect of the secondary metabolite production pathways, since disruption of these pathways decreases the substrate uptake rate, while overexpression of the itaconate production pathway increases it again. Some of the intermediate deletion strains also produced comparatively low amounts of erythritol (Ery; rRT of 0.81) and an unknown compound with rRT of 0.88, indicating that the disruption of major metabolic outlets ‘pushes’ carbon into alternative pathways. Another peak with rRT of 0.62 is apparent in the chromatograms of the wild type and *U. maydis* MB215 ∆UA (Fig. S3). This peak is assumed to be caused by MELs, since it is absent in all strains, which only have the deletion of the MEL cluster in common. The HPLC analysis further confirmed the absence of (*S*)‐2‐hydroxyparaconate (rRT of 0.75) in the *cyp3* deletion mutant. The deletion of all by‐product genes in *U. maydis* MB215 ∆*cyp3* ∆MEL ∆UA ∆*dgat*, resulted in a 1.1‐fold increase in itaconate production compared to the wild type. With the additional overexpression of *ria1* in the final ITA chassis, this increase was 10.2‐fold. The differences in the degree of enhancement between FAMES and HPLC analyses are likely due to C_10_/ITA overlapping in FAMES analysis. Simultaneously to the increase in ITA synthesis, malate (rRT of 0.70) secretion was drastically reduced by 84% due to the upregulation of the mitochondrial antiporter *mtt1* as consequence of the *ria1* overexpression (Jaklitsch *et al.*, 1991; Geiser *et al.*, 2016b).

In the ITA chassis strain, the production of virtually all detectable by‐products could be abolished, while the metabolic flux towards the target product itaconate was drastically increased compared to the wild type.

The deletion of lipidic by‐product formation also turned out to be a major advantage in handling and efficiency of unit operations such as settling or centrifugation. Within minutes of centrifugation, cells of the ITA chassis strain formed a pellet with clear supernatant (Fig. 3D). With the wild type, this effect was not achievable within 60 min. Microscopy images identified strong extracellular lipid production of the wild type (Fig. S2) likely resulting in the formation of a cloudy supernatant of suspended glycolipids during centrifugation. It is also expected that TAG formation affects the density of the cells making a subpopulation neutrally buoyant. Both effects are avoided in the ITA chassis, facilitating downstream processing with this strain.

### Influence of the reduced by‐product spectrum on itaconate production

**Figure 4:**
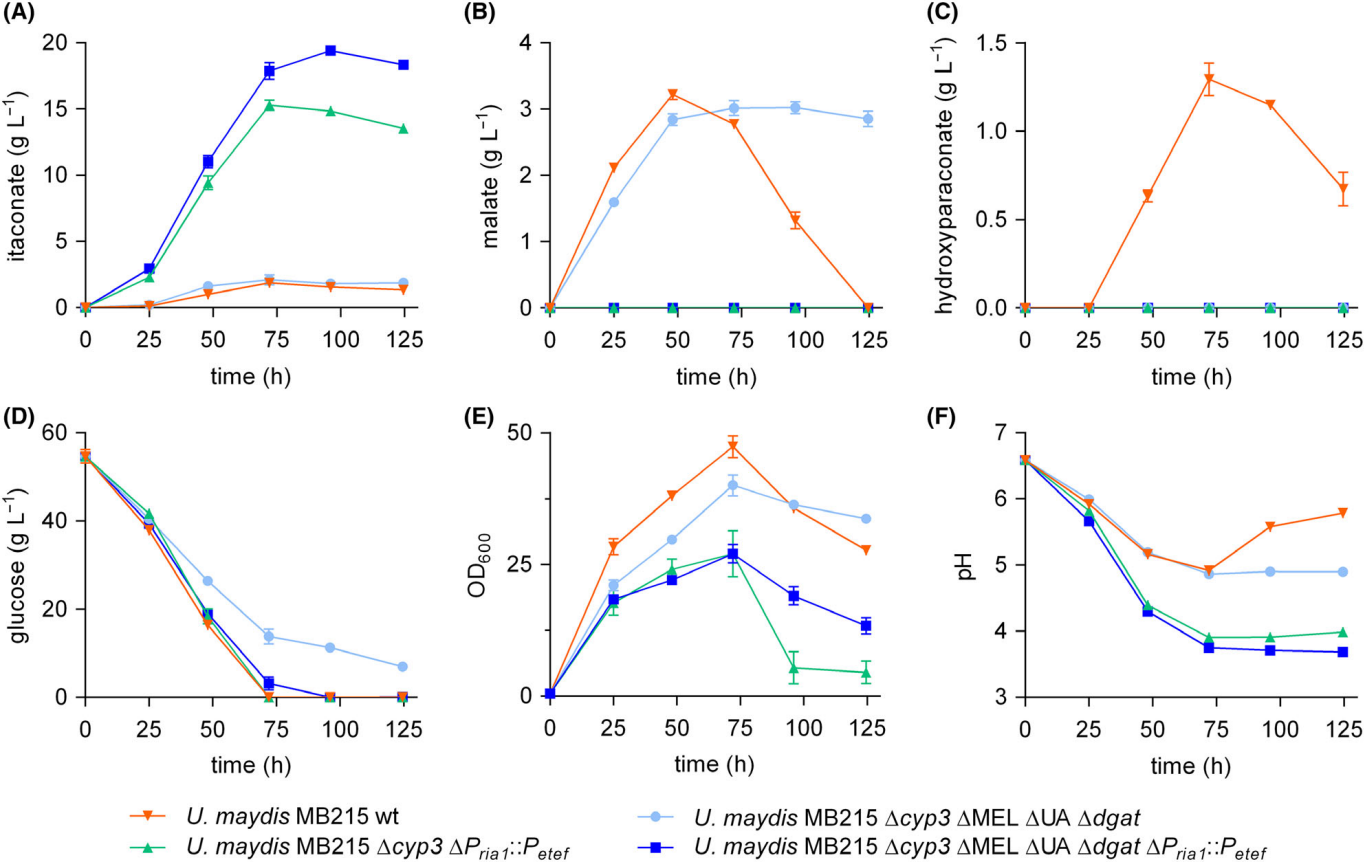
System Duetz^®^ cultivation of various *U. maydis* MB215 strains in screening medium with 50 g l^−1^ glucose and 100 mM MES, incubated in 24‐well plates with a filling volume of 1.5 ml (shaking diameter = 50 mm, n = 300 rpm, T = 30 °C and Φ = 80%). Concentrations of itaconate (A), malate (B), (S)‐2‐hydroxyparaconate (C) and glucose (D) are shown, as well as OD_600_ (E) and pH (F) of cultures of *U. maydis* MB215 wild type (orange, inverted triangle), Δ*cyp3* Δ*P_ria1_*::*P_etef_* (green, triangle), Δ*cyp3* ΔMEL ΔUA Δ*dgat* (light blue, circle) and Δ*cyp3* ΔMEL ΔUA Δ*dgat* Δ*P_ria1_*::*P_etef_* (dark blue, square). Error bars indicate the standard error of the mean (n = 3).

To evaluate the effect of the elimination of by‐product formation on itaconate production in detail, the performance of the *U. maydis* MB215 ITA chassis (∆*cyp3* ∆MEL ∆UA ∆*dgat* ∆*P_ria1_::P_etef_*) and its precursor strain *U. maydis* MB215 ∆*cyp3* ∆MEL ∆UA ∆*dgat* were investigated over time (Fig. 4, Table 1). *U. maydis* MB215 wild type and *U. maydis* MB215 ∆*cyp3* ∆*P_ria1_::P_etef_*, a strain equivalent to the already engineered and published itaconate hyper‐producing strain *U. maydis* MB215 ∆*cyp3 P_etef_ria1* (Geiser *et al.*, 2016b), were included as references. The strains were cultivated in 24‐well System Duetz^®^ plates in screening medium containing an initial concentration of 50 g l^−1^ glucose as sole carbon source buffered with 100 mM MES at a pH of 6.5.

**Table 1:** Production parameters of *U. maydis* MB215 strains cultured in screening medium with 50 g l^−1^ glucose and 100 mM MES, or 100 g l^−1^ glucose and 66 g l^−1^ CaCO_3_. ±values indicate the standard error of the mean (n = 3). Symbols refer to Fig. 4 (MES cultures) or Fig. 5 (CaCO_3_ cultures).

Medium	Symbol	Strain	ITA titre_max_ a (g l^−1^)	q_P_ b (g l^−1^ h^−1^)	q_P,max_ c (g l^−1^ h^−1^)	y_P/S_ d (g_ITA_ g_Glu_^−1^)
50 g l^−1^ glucose + 100 mM MES	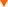	*U. maydis* MB215 wild type	1.9 ± 0.1	0.03 ± 0.00	0.04 ± 0.00	0.03 ± 0.00
	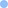	*U. maydis* MB215 ∆*cyp3* ∆MEL ∆UA ∆*dgat*	2.1 ± 0.4	0.02 ± 0.00	0.06 ± 0.00	0.05 ± 0.01
	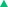	*U. maydis* MB215 ∆*cyp3* ∆*P_ria1_*::*P_etef_*	15.3 ± 0.4	0.21 ± 0.01	0.31 ± 0.02	0.28 ± 0.01
	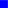	*U. maydis* MB215 ∆*cyp3* ∆MEL ∆UA ∆*dgat* ∆*P_ria1_*::*P_etef_*	19.4 ± 0.3	0.25 ± 0.01	0.35 ± 0.02	0.36 ± 0.02
100 g l^−1^ glucose + 66 g l^−1^ CaCO_3_	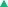	*U. maydis* MB215 ∆*cyp3* ∆*P_ria1_*::*P_etef_*	43.7 ± 4.7	0.22 ± 0.01	0.35 ± 0.03	0.39 ± 0.05
	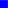	*U. maydis* MB215 ∆*cyp3* ∆MEL ∆UA ∆*dgat* ∆*P_ria1_*::*P_etef_*	53.5 ± 5.0	0.28 ± 0.00	0.39 ± 0.01	0.47 ± 0.03

a. Maximum itaconate titre.

b. Overall itaconate production rate ([glucose] > 3.2 g l^−1^).

c. Maximum itaconate production rate.

d. Yield itaconate per consumed glucose.

The strain with all four by‐product‐forming genes/ gene clusters deleted, but without *ria1* overexpression (*U. maydis* MB215 ∆*cyp3* ∆MEL ∆UA ∆*dgat*) produced 2.1 ± 0.4 g l^−1^ itaconate, which is not significantly different from the wild type. However, *U. maydis* MB215 ∆*cyp3* ∆MEL ∆UA ∆*dgat* consumed 25% less glucose than the wild type resulting in a 51% higher product to substrate yield (y_p/s_) for itaconate from glucose. This confirms the previous observations which suggest that this strain lacks sufficient metabolic ‘outlet’, thereby reducing its substrate uptake rate, when growth as predominant C‐sink is no longer possible under the applied nitrogen limitation.

The itaconate producing strain *U. maydis* MB215 ∆*cyp3* ∆*P_ria1_::P_etef_* achieved a maximum titre of 15.3 ± 0.4 g l^−1^ itaconate, which is 8.1‐fold higher than that of the wild type. With the ITA chassis, the itaconate titre was further increased to 19.4 ± 0.3 g l^−1^ constituting a 10.2‐fold increase compared to the wild type and accordingly a 1.3‐fold increase compared to *U. maydis* MB215 ∆*cyp3* ∆*P_ria1_*::*P_etef_*. The OD_600_ of the ITA chassis dropped significantly towards the end of the culture, likely caused by a change in morphology (Hosseinpour Tehrani *et al.*, 2019c). The maximum volumetric production rate (q_p,max_) was increased by 13%, while the overall rate (q_p_) was increased by 19%, and the y_p/s_ by 29% compared to the latter strain. These results show that the four deletions reduce the metabolic flux of glucose into these by‐product pathways, reflected in a decreased glucose uptake rate. The fact that this alone doesn't lead to a much higher product titre clearly indicates a bottleneck at the level of the itaconate production pathway, which was eliminated by overexpression of *ria1* resulting in an upregulation of itaconate cluster genes (Geiser *et al.*, 2016c). The combination of the four knockouts and ∆*P_ria1_::P_etef_* led to a significant improvement in the itaconate titre and the corresponding rate and yield parameters. The direct comparison of *U. maydis* MB215 ∆*cyp3* ∆*P_ria1_*::*P_etef_* and the ITA chassis verified the added benefit of the lipid knockouts on itaconate production.

**Figure 5:**
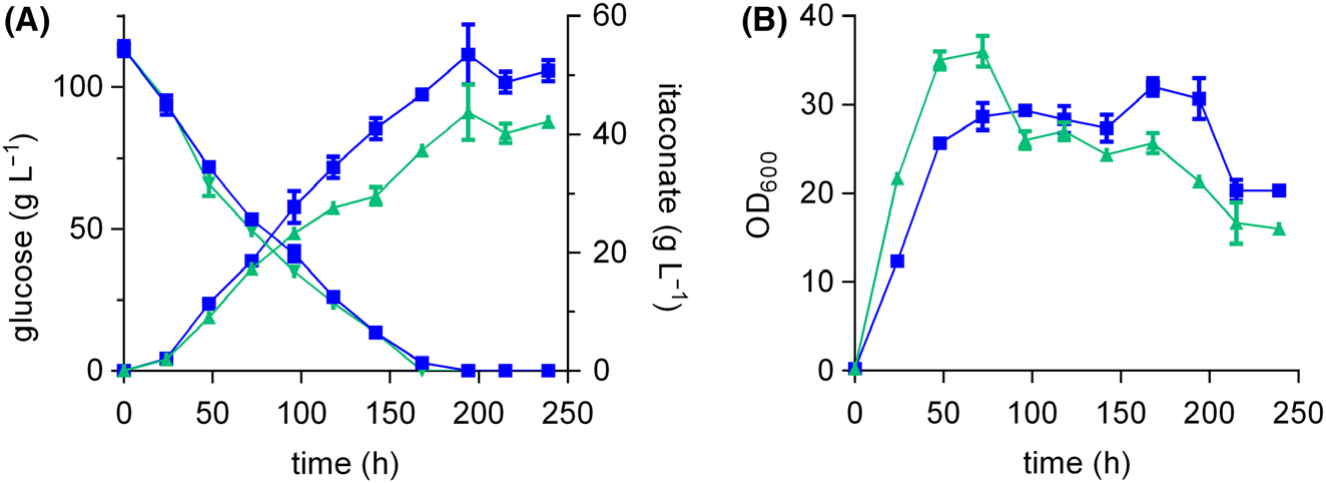
Shake flask cultivation of two *U. maydis* MB215 mutant strains in screening medium with 100 g l^−1^ glucose and 66 g l^−1^ CaCO_3_, incubated in 500 ml Erlenmeyer flasks with a filling volume of 50 ml (shaking diameter = 25 mm, n = 200 rpm, T = 30 °C and Φ = 80%). Itaconate production and glucose consumption (A) and OD_600_ (B) of *U. maydis* MB215 ∆*cyp3* ∆*P_ria1_::P_etef_* (green, triangle) and *U. maydis* MB215 ∆*cyp3* ∆MEL ∆UA ∆*dgat* ∆*P_ria1_*::*P_etef_* (blue, square). Error bars indicate the standard error of the mean (*n* = 3).

The production of large amounts of itaconate goes along with a drastic drop of the pH value in the medium. In the previous experiment, the pH decreased from 6.5 to 3.68 for the ITA chassis. This likely affects itaconate productivity as *U. maydis* is not particularly tolerant to low pH (Hosseinpour Tehrani *et al.*, 2019b). In order to eliminate this potential pH inhibition, the buffer system was changed from MES to CaCO_3_ and the substrate concentration was increased. The use of CaCO_3_ as insoluble buffer enables a much larger buffer capacity and further provides an *in situ* product sink, as calcium itaconate is only soluble up to 14.5 g l^−1^. It does however pose its own process‐associated challenges such as high solids loads (Hosseinpour Tehrani *et al.*, 2019a). The much higher buffer capacity of 66 g l^−1^ CaCO_3_ keeps the pH constant between 6 and 7, even with an increased initial glucose concentrations of 100 g l^−1^ (Fig. 5A). From 100 g l^−1^ fully consumed glucose, *U. maydis* MB215 ∆*cyp3* ∆*P_ria1_::P_etef_* produced a maximum of 43.7 ± 4.7 g l^−1^ itaconate within 194 h corresponding to a y_p/s_ of 0.39 ± 0.05 g_ITA_ g_Glu_^−1^ and q_p_ of 0.22 ± 0.01 g l^−1^ h^−1^ (Fig. 5A; Table 1). In the same time and with comparable glucose consumption behaviour, the ITA chassis reached a titre of 53.5 ± 5.0 g l^−1^ accomplishing an y_p/s_ of 0.47 ± 0.03 g_ITA_ g_Glu_^−1^ and q_p_ of 0.28 ± 0.00 g l^−1^ h^−1^. These parameters confirm that the three by‐product deletions ∆MEL, ∆UA and ∆*dgat* resulted in a more efficient conversion of glucose into itaconate and therefore in a 21 – 27% improvement of itaconate yield, titre and rate (Table 1). The same trend was observed with an initial glucose concentration of 150 g l^−1^ (Fig. S4; Table S1), but with slightly lower yields and production rates likely due to higher osmotic stress.

## Conclusions

This study focused on the reduction of the *U. maydis* metabolite spectrum to improve its itaconate production. The elimination of by‐product formation significantly enhanced itaconate titre, rate, and yield. In addition, the lack by‐products, and better handling of the strain, will contribute to a more efficient downstream processing. This is particularly crucial for itaconate applications in the polymer industry, where high purities are generally required. With its optimized performance, the designed strain lays the basis for further process optimizations such as fed‐batch fermentations and cell retention. In addition, upon removal of the itaconate gene cluster, it's clean product background and marker‐free genome makes the *U. maydis* MB215 ITA chassis an ideal platform for the synthesis of a variety of other industrially relevant products such as malate or erythritol.

## Experimental procedures

### Media and culture conditions

*Escherichia coli* strains were grown in medium containing 10 g l^−1^ peptone, 5 g l^−1^ sodium chloride, 5 g l^−1^ yeast extract and 5 g l^−1^ glucose. *U. maydis* strains were grown in YEPS medium containing 10 g l^−1^ yeast extract, 10 g l^−1^ peptone and 10 g l^−1^ sucrose. As screening medium for production experiments, *U. maydis* was cultivated in modified Tabuchi medium (MTM) according to Geiser *et al.* (2014). Besides varying glucose and buffer (2‐(N‐morpholino) ethanesulfonic acid (MES; pH adjusted to 6.5 with NaOH) or CaCO_3_) concentrations, this medium contained 0.8 g l^−1^ NH_4_Cl, 0.2 g l^−1^ MgSO_4_·7H_2_O, 0.01 g l^−1^ FeSO_4_·7H_2_O, 0.5 g l^−1^ KH_2_PO_4_, 1 ml l^−1^ vitamin solution and 1 ml l^−1^ trace element solution. The vitamin solution contained (per litre) 0.05 g D‐biotin, 1 g D‐calcium pantothenate, 1 g nicotinic acid, 25 g myo‐inositol, 1 g thiamine hydrochloride, 1 g pyridoxol hydrochloride and 0.2 g para‐aminobenzoic acid. The trace element solution contained (per litre) 1.5 g EDTA, 0.45 g ZnSO_4_∙7H_2_O, 0.10 g MnCl_2_∙4H_2_O, 0.03 g CoCl_2_∙6H_2_O, 0.03 g CuSO_4_∙5H_2_O, 0.04 g Na_2_MoO_4_∙2H_2_O, 0.45 g CaCl_2_∙2H_2_O, 0.3 g FeSO_4_∙7H_2_O, 0.10 g H_3_BO_3_ and 0.01 g KI. When using CaCO_3,_ medium components were added relative to the total volume of solids + liquid, leading to a higher aqueous concentration of soluble components. Shaking cultures of *U. maydis* were performed in 24‐well System Duetz^®^ plates with a filling volume of 1.5 ml (shaking diameter = 50 mm, n = 300 rpm, T = 30 °C and Φ = 80%) (Duetz *et al.*, 2000) or in 500 ml shaking flasks with a filling volume of 50 ml (shaking diameter = 25 mm, n = 200 rpm, T = 30 °C and Φ = 80%). When using System Duetz^®^, cultures were inoculated in parallel into multiple plates in order to ensure continuous oxygenation by taking a complete plate as sacrificial sample for each sample point (Table 2).

**Table 2:** *Ustilago maydis* MB215 strains used in this study.

Strain designation	Resistance	Reference
*U. maydis* MB215		Hewald *et al.* (2005)
*U. maydis* MB215 ∆*cyp3* ∆*P_ria1_*::*P_etef_*		Hosseinpour Tehrani *et al.* (2019a)
*U. maydis* MB215 ∆*cyp1*	hyg^R^	Hewald *et al.* (2005)
*U. maydis* MB215 ∆*cyp1* ∆*emt1*	hyg^R^	Hewald *et al.* (2005)
*U. maydis* MB215 ∆*cyp3*		This study
*U. maydis* MB215 ∆*emt1*		This study
*U. maydis* MB215 ∆UA		This study
*U. maydis* MB215 ∆MEL		This study
*U. maydis* MB215 ∆*cyp3* ∆MEL		This study
*U. maydis* MB215 ∆*cyp3* ∆MEL ∆UA		This study
*U. maydis* MB215 ∆*cyp3* ∆MEL ∆UA ∆*dgat*		This study
*U. maydis* MB215 ∆*cyp3* ∆MEL ∆UA ∆*dgat* ∆*P_ria1_*::*P_etef_* (=ITA chassis)		This study

### Analytical methods

All values are the arithmetic mean of at least three biological replicates. Error bars or ± values indicate the standard error of the mean.

When using CaCO_3_ as buffer, 1 ml culture broth was taken for OD_600_ and HPLC analysis. The CaCO_3_ was dissolved 1:1 with 4 M HCl prior to further measurements as described in (Zambanini *et al.*, 2016). Cell densities were measured by determining the absorption at 600 nm with an Ultrospec 10 Cell Density Meter (Amersham Biosciences, Chalfont Saint Giles, UK).

For thin‐layer chromatography (TLC) analysis of mannosylerythritol lipids and ustilagic acid, the *U. maydis* strains were cultivated for 72 h in yeast nitrogen base/5% glucose medium. 0.3 ml culture was mixed with 0.6 ml ethyl acetate for 20 min. After centrifugation (5 min, 14 000 g), the ethyl acetate phase was dried overnight at room temperature (RT). The residue was resuspended in 50 µl methanol, whereof 6 µl were spotted on a TLC Silica gel 60 aluminium sheet (20 x 20 cm, Merck KGaA, Darmstadt, Germany) and dried for 5 min at RT. As running buffer, a mixture of chloroform, methanol and H_2_O (70:26:4, v/v) was used. The plate was dried for 10 min at RT, before glycolipids were visualized by staining with a mixture of acetic acid, *p*‐anisaldehyde and sulphuric acid (97:1:2, v/v) followed by heating at 120 °C for 3–5 min.

For fatty acid methyl esters (FAMES) analysis, lipid extracts were obtained from 9 ml culture broth after 72 h of cultivation in MTM (50 g l^−1^ glucose, pH 6.5 buffered with 100 mM MES, System Duetz^®^). Lipid composition was analysed by lipid hydrolysis, esterification to fatty acid methyl esters using methanolic H_2_SO_4_ as transmethylating reagent and hexane extraction. GC‐FID separation was performed in a Shimadzu GC‐MS QP 2010 using an OPTIMA^®^ 225 column (30 m, 0.25 mm, 0.25 µm; Macherey‐Nagel, Germany).

For high‐performance liquid chromatography (HPLC) analysis, all samples were filtered with Rotilabo^®^ (CA, 0.2 µm, Ø 15 mm) or Acrodisc^®^ Syringe Filters (GHP, 0.2 µm, Ø 13 mm) and diluted 1:10 with 5 mM H_2_SO_4_. Products in the supernatant were analysed in a DIONEX UltiMate 3000 HPLC System (Thermo Scientific, Langerwehe, Germany) with an ISERA Metab‐AAC column 300 × 7.8 mm column (ISERA, Germany). As solvent, 5 mM H_2_SO_4_ with a constant flow rate of 0.6 ml min^−1^ and a temperature of 40 °C was used. For detection, a SHODEX RI‐101 detector (Showa Denko Europe GmbH, Munich, Germany) and a DIONEX UltiMate 3000 Variable Wavelength Detector set to 210 nm were used. Analytes were identified via retention time and UV/RI quotient compared to corresponding standards. For optimal comparison of HPLC chromatograms, retention times were specified relative to the retention time of itaconate. With this normalization, small analytical variations are minimized, enabling a more accurate comparison of overlapping peaks in the start of the chromatogram.

For genome re‐sequencing, genomic DNA was isolated by phenol‐chloroform‐isoamyl alcohol extraction. Whole‐genome sequencing was performed by GATC Biotech AG (Konstanz, Germany) using Genome Sequencer Illumina HiSeq 2500 technology (sequence mode: 2 × 150 bp read length) and the HiSeq Control Software v2.0.12.0 (Illumina Inc., San Diego, CA, USA). Variance analysis in terms of SNP and InDel detection was conducted by mapping the re‐sequencing data onto the *U. maydis* 521 reference genome (Kämper *et al.*, 2006; Refseq assembly GCF_000328475.2). Mapping and variant calling results were visualized with Integrative Genomics Viewer (IGV) software (Thorvaldsdottir *et al.*, 2013). The sequencing data are available through the NCBI Sequence Read Archive (PRJNA592070).

### Plasmid cloning and strain engineering

Plasmids were constructed by Gibson assembly (Gibson *et al.*, 2009) using the NEBuilder^®^ HiFi DNA Assembly Cloning Kit (New England Biolabs (NEB), Ipswich, MA, USA). Primers were ordered as DNA oligonucleotides from Eurofins Genomics (Ebersberg, Germany). As polymerase, Q5^®^ High‐Fidelity DNA Polymerase (NEB) was used. Detailed information about utilized primers and plasmids are listed in Tables S2 and S3. Competent *E. coli* DH5α were used for standard cloning and plasmid maintenance according to Sambrook and Russell (2001). Plasmids were confirmed by PCR or sequencing. Generation of protoplasts and transformation of *U. maydis* were performed according to (Brachmann *et al.* (2004). Genomic DNA of *U. maydis* was isolated according to Hoffman and Winston (1987). For the deletion of *cyp3*, MEL and UA gene cluster, and for the exchange of the native *ria1* promoter with the strong constitutive *P_etef_*, the CRISPR/Cas9 system (Schuster *et al.*, 2016) in combination with repair templates was used. The repair templates consisted of two 1000 bp long fragments corresponding to the flanking regions upstream and downstream of the sequence to be eliminated. For the deletion of *dgat*, homologous recombination with 1000 bp flanking regions including FRT‐sites and a hygromycin resistance cassette were used (Khrunyk *et al.*, 2010). Successful integration and deletion were verified by PCR and sequencing.

## Conflict of interest

None declared.

## Author contributions

All authors contributed significantly to the work. NW conceived the study. JL designed and performed experiments and analysed results with the help of NW, HHT and LMB. MG and JM performed FAMES lipid analysis. JL wrote the manuscript with help of NW and LMB.

## Supporting information


**Fig. S1.** Genomic sequence after exchange of the native *ria1* promoter by the constitutive *etef* promoter encoding gene.

**Fig. S2.** Identification of mannosylerythritol lipid (white arrow) and ustilagic acid (black arrow) production in *U. maydis* MB215 wildtype (left) and *U. maydis* MB215 ITA chassis (right) by microscopy.

**Fig. S3.** FAMES and HPLC diagrams of different *U. maydis* MB215 mutant strains.

**Fig. S4.** Shake flask cultivation of two *U. maydis* MB215 mutant strains in screening medium.

**Table S1.** Production parameters of two engineered *U. maydis* MB215 strains resulting from a cultivation in screening medium.
**Table S2.** Plasmids used in this study.
**Table S3.** Oligonucleotides used for deletion and overexpression constructs.
