# Supplementary figures and images for: An Ustilago maydis chassis for itaconic acid production without by‐products
Source: Microb Biotechnol. 2019 Dec 27;13(2):350–62. doi: 10.1111/1751-7915.13525 (PMC7017832; doi:10.1111/1751-7915.13525)

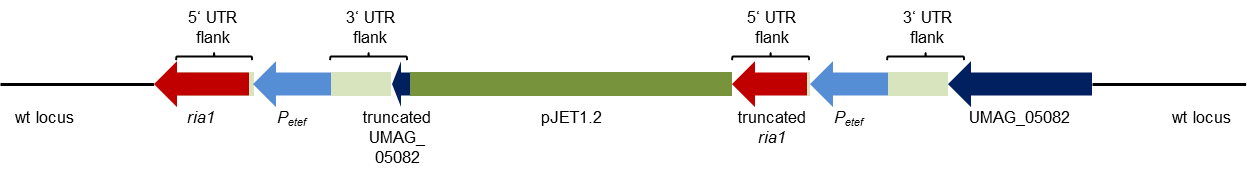

Supplement: Supplementary file 1 — Fig. S1. Genomic sequence after exchange of the native ria1 promoter by the constitutive etef promoter encoding gene. [file MBT2-13-350-s001.tif]

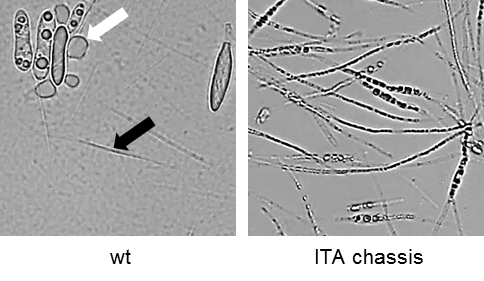

Supplement: Supplementary file 2 — Fig. S2. Identification of mannosylerythritol lipid (white arrow) and ustilagic acid (black arrow) production in U. maydis MB215 wildtype (left) and U. maydis MB215 ITA chassis (right) by microscopy. [file MBT2-13-350-s002.tif]
