# Supplementary material for: An Ustilago maydis chassis for itaconic acid production without by‐products
Source: Microb Biotechnol. 2019 Dec 27;13(2):350–62. doi: 10.1111/1751-7915.13525 (PMC7017832; doi:10.1111/1751-7915.13525)
Supplement: Supplementary file 5 — Table S1. Production parameters of two engineered U. maydis MB215 strains resulting from a cultivation in screening medium. Table S2. Plasmids used in this study. Table S3. Oligonucleotides used for deletion and overexpression constructs. [file MBT2-13-350-s005.docx]

1. Supplement

**Fig. S1**


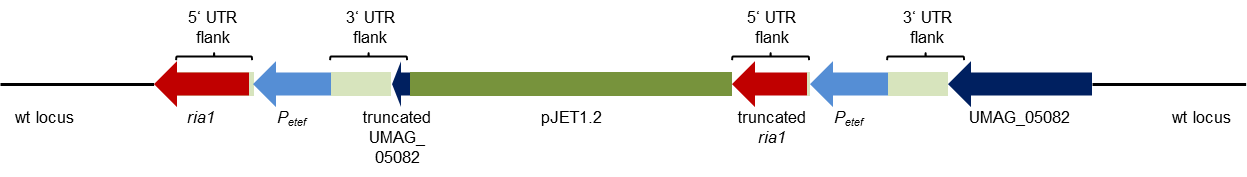


Fig. S1. Genomic sequence after exchange of the native *ria1* promoter by the constitutive *etef* promoter encoding gene.

**Fig. S2**


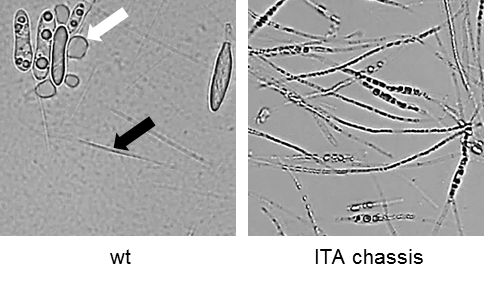


Fig. S2. Identification of mannosylerythritol lipid (white arrow) and ustilagic acid (black arrow) production in *U. maydis* MB215 wildtype (left) and *U. maydis* MB215 ITA chassis (right) by microscopy.

**Fig. S3**





Fig. S3. FAMES and HPLC diagrams of different *U. maydis* MB215 mutant strains. For HPLC analysis, supernatants were obtained from culture broths after 96 h (*U. maydis* MB215 ∆MEL, ∆*cyp3* ∆MEL, and ∆*cyp3* ∆MEL ∆UA) and 124.5 h (*U. maydis* MB215 ∆*cyp3* ∆MEL ∆UA ∆*dgat*) of cultivation in screening medium with 50 g L^-1^ glucose and a pH 6.5 buffered with 100 mM MES using 24-well System Duetz^®^ plates. Retention times are shown relative to itaconic acid. Glu: glucose, MEL: mannosylerythritol lipids, Mal: malate, 2-HP: (S)-2-hydroxyparaconate, Ery: erythritol, ITA: itaconate. For FAMES analysis, all strains were cultivated for 72 h in screening medium with 50 g L^-1^ glucose and a pH 6.5 buffered with 100 mM MES using 24-well System Duetz^®^ plates. Retention time correlates with chain length.

**Fig. S4**





Fig. S4. Shake flask cultivation of two *U. maydis* MB215 mutant strains in screening medium.

Itaconate production and glucose consumption (A) and OD_600_ (B) measured over time for *U. maydis* MB215 ∆*cyp3* ∆*P_ria1_::P_etef_* (green) and *U. maydis* MB215 ∆*cyp3* ∆MEL ∆UA ∆*dgat* ∆*P_ria1_*::*P_etef_* (blue) cultivated in screening medium containing 150 g L^-1^ glucose and 99 g L^-1^ CaCO_3_ as pH 6.5 buffer. Error bars indicate the standard error of the mean (n = 3).

**Tab. S1**

Tab. S1. Production parameters of two engineered *U. maydis* MB215 strains resulting from a cultivation in screening medium.

*U. maydis* MB215 ∆*cyp3* ∆*P_ria1_*::*P_etef_* (green) and *U. maydis* MB215 ∆*cyp3* ∆MEL ∆UA ∆*dgat* ∆*P_ria1_*::*P_etef_* (dark blue) were screened in screening medium containing 150 g L^-1^ glucose + 99 g L^-1^ CaCO_3_. ±values indicate the standard error of the mean (n = 3).

| **medium** | **symbol** | **strain** | **ITA titer_max_^*^**  **(g L^-1^)** | **q_P_^**^**  **(g L^-1^ h^-1^)** | **q_P,max_^***^**  **(g L^-1^ h^-1^)** | **y_P/S_^****^**  **(g_ITA_ g_Glu_^-1^)** |
| --- | --- | --- | --- | --- | --- | --- |
| 150 g L^-1^ glucose  +  99 g L^-1^ CaCO_3_ | ▲ | *U. maydis* MB215 ∆*cyp3* ∆*P_ria1_* ::*P_etef_* | 50.9 ± 1.2 | 0.21 ± 0.00 | 0.30 ± 0.00 | 0.38 ± 0.01 |
|  | ■ | *U.* *maydis* MB215 ∆*cyp3* ∆MEL ∆UA ∆*dgat* ∆*P_ria1_* ::*P_etef_* | 55.7 ± 1.7 | 0.23 ± 0.01 | 0.36 ± 0.02 | - 1. 0.01 |
| * maximum itaconic acid titer ** overall itaconate production rate ([glucose] > 3.2 g L^-1^)  *** maximum itaconate production rate **** yield itaconate per glucose consumed | | | | | | |

**Tab. S2**

Tab. S2. Plasmids used in this study.

| **plasmid** | **description** | **reference** |
| --- | --- | --- |
| pJET1.2/blunt | Ori ColE1; AmpR | Thermo Scientific, Germany |
| pJET1.2-*ura*-sgRNA | pJET1.2 with sgRNA of *ura* for CRISPR/Cas9 modifications | this study |
| pMS8-Cas9 | U6 promoter; constitutive *otef* promoter; codon-optimized *cas9* from *Streptococcus pyogenes*; *ip*^R^; ARS; ampR; | Prof. R. Kahmann, Philipps University Marburg, Germany |
| pFLPexpC | *Pcrg1* promoter; synthetic *FLP recombinase* gene; cbxR; ARS; AmpR | Prof. M. Feldbrügge, Heinrich-Heine University Düsseldorf, Germany |
| pStorI-1rh wt | FRTwt-HygR-FRTwt cassette; GentR | Prof. M. Feldbrügge, Heinrich-Heine University Düsseldorf, Germany |
| pJET1.2-*cyp3*-donor template | pJET1.2 with 5’- and 3’-UTR flank of UMAG_05074 as deletion construct | this study |
| pJET1.2-*cyp3*-sgRNA | pJET1.2 with sgRNA of UMAG_05074 for CRISPR/Cas9 modifications | this study |
| pMS8-*cyp3*-sgRNA | pMS8-Cas9 with sgRNA of UMAG_05074 for CRISPR/Cas9 modifications | this study |
| pJET1.2-MEL cluster-donor template | pJET1.2 with 5’-UTR flank of UMAG_03114 and 3’-UTR flank of UMAG_10636 as deletion construct | this study |
| pJET1.2-*emt1*-donor template | pJET1.2 with ampR; 5’- and 3’-UTR flank of UMAG_03117 | this study |
| pJET1.2-*emt1*-sgRNA | pJET1.2 with sgRNA of UMAG_03117 for CRISPR/Cas9 modifications | this study |
| pMS8-*emt1*-sgRNA | pMS8-Cas9 with sgRNA of UMAG_03117 for CRISPR/Cas9 modifications | this study |
| pJET1.2-UA cluster-donor template | pJET1.2 with 5’-UTR flank of UMAG_96458 and 3’-UTR flank of UMAG_12340 as deletion construct | this study |
| pJET1.2-*cyp1*-donor template | pJET1.2 with 5’- and 3’-UTR flank of UMAG_06463 as deletion construct | this study |
| pJET1.2-*cyp1*-sgRNA | pJET1.2 with sgRNA of UMAG_06463 for CRISPR/Cas9 modifications | this study |
| pMS8-*cyp1*-sgRNA | pMS8-Cas9 with sgRNA of UMAG_06463 for CRISPR/Cas9 modifications | this study |
| pJET1.2-*dgat* 5’-UTR flank -FRTwt-HygR-FRTwt-dgat 3’-UTR flank | pJET1.2 with 5’- and 3’-UTR flank of UMAG_03937 as deletion construct; HygR; FRT wt recombination sites | this study |
| pMS8-Um_*P_oma_*-sgRNA | pMS8-Cas9 with sgRNA of *P_oma_* promoter for CRISPR/Cas9 modifications | this study |
| pJET1.2-Um_*P_etef_* -donor template | pJET1.2 with cDNA of *P_etef_* promoter as deletion construct | this study |

**Tab. S3**

Tab. S3. Oligonucleotides used for deletion and overexpression constructs.

| **primer name** | **sequence (5’-3’) & description** |
| --- | --- |
| JB-1_fwd | AGATGGCGTTGCCAGAATCGGTTTTAGAGCTAGAA  Amplification of pJET1.2-*ura*-sgRNA to change *ura*-sgRNA by *cyp3*-sgRNA |
| JB-2_fwd | ctcgagtttttcagcaagatGTAGACGAAAGCCACCTG  Amplification of 5’-UTR flank for *cyp3* donor template construction |
| JB-3_rev | catcggtgtcGATGCTCCCAAACGCCTC  Amplification of 5’-UTR flank for *cyp3* donor template construction |
| JB-4_fwd | tgggagcatcGACACCGATGCAGCCGCA  Amplification of 3’-UTR flank for *cyp3* donor template construction |
| JB-5_rev | aggagatcttctagaaagatTCGGCTTCCACCCGCTTG  Amplification of 3’-UTR flank for *cyp3* donor template construction |
| JB-6_fwd | CCACCTGCGTGTACAGCTTG  Amplification of *cyp3* donor template |
| JB-7_rev | CAAGTCGTGAGTCGGACAAG  Amplification of *cyp3* donor template |
| JB-8_fwd | CAAAATTCCATTCTACAACGAGAT  Amplification of *cyp3*-sgRNA for insertion into pMS8-Cas9 |
| JB-9_fwd | CTTCAAGGCCACCACAAC  Verification of *cyp3* deletion |
| JB-64_rev | TGCTGCCATGGTGCTACTCC  Verification of *cyp3* deletion |
| JB-11_fwd | TCAGCTCGTACGCCGTCGCTGTTTTAGAGCTAGAA  Amplification of pJET1.2-*ura*-sgRNA to change *ura*-sgRNA by *emt1*-sgRNA |
| JB-12_fwd | CAAAATTCCATTCTACAACGTCAG  Amplification of *emt1*-sgRNA for insertion into pMS8-Cas9 |
| JB-13_fwd | ctcgagtttttcagcaagatGACGAGGCTTAGCTAGTC  Amplification of 5’-UTR flank for *emt1* donor template construction |
| JB-14_rev | taacaccatcCCTCGACACTCACGACTC  Amplification of 5’-UTR flank for *emt1* donor template construction |
| JB-15_fwd | agtgtcgaggGATGGTGTTAGGTTCCGAG  Amplification of 3’-UTR flank for *emt1* donor template construction |
| JB-16_rev | aggagatcttctagaaagatCTGTGGCACGTCTCTGAT  Amplification of 3’-UTR flank for *emt1* donor template construction |
| JB-17_fwd | CTAGCCTGAGAAGCTCTATC  Amplification of *emt1* donor template |
| JB-18_rev | GTGAATGGTTCATGGATGGC  Amplification of *emt1* donor template |
| JB-19_fwd | GACGAGGAGGTGTGGATTTC  Verification of *emt1* deletion |
| JB-20_rev | GAGTACCAGATTCGCCAGCT  Verification of *emt1* deletion |
| JB-21_fwd | ctcgagtttttcagcaagatCGCATTTGCTCACATGTATCGC  Amplification of 5’-UTR flank of *mat1* for MEL gene cluster donor template construction |
| JB-22_rev | cagcaatgggCAGGCCAAGCTATGGCCG  Amplification of 5’-UTR flank of *mat1* for MEL gene cluster donor template construction |
| JB-23_fwd | gcttggcctgCCCATTGCTGTCACTCTC  Amplification of 3’-UTR flank of *mac2* for MEL gene cluster donor template construction |
| JB-24_rev | aggagatcttctagaaagatGACTCCCAACTGTGGGCA  Amplification of 3’-UTR flank of *mac2* for MEL gene cluster donor template construction |
| JB-25_fwd | GTTGCAGTTCCAAGCTAACG  Amplification of MEL cluster donor template |
| JB-26_rev | CGATTGCGGTGAAGGGATGC  Amplification of MEL cluster donor template |
| JB-28_rev | GTCAGTCGTGAGTGTTAAGG  Verification of MEL gene cluster deletion |
| JB-29_fwd | TCACGGTTCCCTCTGTTATC  Verification of MEL gene cluster deletion |
| JB-41_fwd | ACGCGTCGTCAGTGTAAATG  Verification of MEL gene cluster deletion |
| JB-42_rev | ACCCGTCCAGCACAGTATCCA  Verification of MEL gene cluster deletion |
| JB-65_fwd | GCCCACCACTCACCACTTTC  Verification of MEL cluster deletion |
| JB-66_rev | ACACCACCGATAGTCCATTG  Verification of MEL cluster deletion |
| JB-31_fwd | TCTGCATGTACGAGAAAAGCGTTTTAGAGCTAGAA  Amplification of pJET1.2-*ura*-sgRNA to change *ura*-sgRNA by *cyp1*-sgRNA |
| JB-38_fwd | CAAAATTCCATTCTACAACGTCTG  Amplification of *cyp1*-sgRNA for insertion into pMS8-Cas9 |
| JB-43_fwd | ctcgagtttttcagcaagatGATTCCATCCGAAGCTGC  Amplification of 5’-UTR flank of *rua1* for UA gene cluster donor template construction |
| JB-44_rev | caattttcacGACGGAAGATGAGAATCG  Amplification of 5’-UTR flank of *rua1* for UA gene cluster donor template construction |
| JB-45_fwd | atcttccgtcGTGAAAATTGGGGCCAATGAC  Amplification of 3’-UTR flank of *ahd1* for MEL gene cluster donor template construction |
| JB-46_rev | aggagatcttctagaaagatCGATTGCCTTTGCCGTGC  Amplification of 3’-UTR flank of *ahd1* for MEL gene cluster donor template construction |
| JB-47_fwd | GCTTCTCGGCTGACGACAAC  Amplification of UA cluster donor template |
| JB-48_rev | GCCATTTCGCCTGTGTACTC  Amplification of UA cluster donor template |
| JB-50_rev | GCCATTTCGCCTGTGTACTC  Verification of UA cluster deletion |
| JB-51_rev | TGAGGGCAATTGTTGTCCTG  Verification of UA cluster deletion |
| JB-67_fwd | GAGCCAGTCAGTCAACAATC  Verification of UA gene cluster deletion |
| JB-53_fwd | ctcgagtttttcagcaagatGTTGATGCCGGTGTGGTTG  Amplification of 5’-UTR flank for generation of *dga*t deletion construct |
| JB-56_rev | aggagatcttctagaaagatCGAACAACACAGTCAAGATGTC  Amplification of 3’-UTR flank for generation of *dgat* deletion construct |
| JB-84_rev | acttctggccGGCTCGTCTGAAGGGTCG  Amplification of 5’-UTR flank for generation of *dgat* deletion construct |
| JB-85_fwd | cagacgagccGGCCAGAAGTTCCTATTC  Amplification of FRT_wt-HygR-FRT_wt cassette for generation of *dgat* deletion construct |
| JB-86_rev | gcacatatcgGGCCAGAAGTTCCTATAC  Amplification of FRT_wt-HygR-FRT_wt cassette for generation of *dgat* deletion construct |
| JB-87_fwd | acttctggccCGATATGTGCGGGTGCAG  Amplification of 3’-UTR flank for generation of *dgat* deletion construct |
| JB-58_fwd | CGCTTGCTGCCAGCTGATTG  Amplification of *dgat* deletion construct |
| JB-59_rev | AGCATACCGCCACCCTCTTG  Amplification of *dgat* deletion construct |
| JB-60_fwd | TCTCTACCTTTGCCGATCTG  Verification of *dgat* deletion |
| JB-61_rev | GTGCAGGTGGTTTGGATTTG  Verification of *dgat* deletion |
| JB-68_fwd | GAAGTGGCTGAGTCGTGTAG  Verification of *dgat* deletion |
| JB-69_rev | ACAGACTGCCAGTCACATTC  Verification of *dgat* deletion |
| HT-4a_rev | ACAGACGTCGCGGTGAGTTC  Verification of FRT-HygR-cassette based insertions |
| HT-8a_rev | GTCGAGCTCGGTACGGGT  Amplification of target sgRNA from pJET1.2 backbone for insertion into pMS8-Cas9 |
| HT-9_fwd | CCTTGCAATTCGCGCACACC  Verification of target-sgRNA insertion into pMS8-Cas9 |
| HT-10_rev | GCTCGGTACGGGTACTAATG  Verification of target-sgRNA insertion into pMS8-Cas9 |
| HT-12_rev | CGTTGTAGAATGGAATTTTG  Amplification of pJET1.2-*ura*-sgRNA to change *ura*-sgRNA by target-sgRNA |
| HT-125_fwd | ATGTCGAGGCCAACTGTG  Amplification of *pETEF* promoter upstream of *ria1* |
| HT-127_rev | TCGAGCCAAATCAATGCG  Amplification of *pETEF* promoter upstream of *ria1* |
| HT-153_fwd | GACAGCGCCCTTTATTGG  Verification of *pOMA* to *pETEF* promoter exchange upstream of *ria1* |
| HT-159_fwd | GCCCAACTGATTAGCTGTGCCCCCTCGC  Verification of pJET1.2-Um_*pETEF*-F-donor template upstream of *ria1* |
| HT-186_rev | AGTTGGGTTCGCTCGATG  Verification of *P_oma_* to *P_etef_* promoter exchange upstream of *ria1* |
